# Non-elective and revision arthroplasty are independently associated with hip and knee prosthetic joint infection caused by *Acinetobacter baumannii*: a Brazilian single center observational cohort study of 98 patients

**DOI:** 10.1186/s12891-021-04393-4

**Published:** 2021-06-02

**Authors:** Raquel Bandeira da Silva, Rodrigo Otavio Araujo, Mauro José Salles

**Affiliations:** 1 Department of Internal Medicine Hospital São Francisco de Assis, Belo Horizonte, MG Brazil; 2 Department of Orthopedics Hospital São Francisco de Assis, Belo Horizonte, MG Brazil; 3 Division of Infectious Diseases, Santa Casa de São Paulo School of Medical Sciences, São Paulo, Brazil; 4 Departamento de medicina, Universidade Federal de São Paulo- Escola Paulista de Medicina (UNIFESP-EPM), Laboratório LEMC, Disciplina de infectologia, São Paulo, SP Brazil

## Abstract

**Background:**

Prosthetic joint infection (PJI) caused by *Acinetobacter baumannii* (*Ab*) has become a growing concern due to its overwhelming ability to express resistance to antibiotics and produce biofilm.

**Aim:**

This study aimed to identify independent risk factors (RFs) associated with *Ab*-associated PJI and their role in the treatment outcome.

**Methods:**

This was a single-centre, retrospective cohort study of PJI patients diagnosed between January 2014 and July 2018. A PJI diagnosis was made based upon the MSIS 2018 criteria. To estimate RFs associated with *Ab*-associated PJI, multivariate analyses with a level of significance of *p* < 0.05 were performed. To evaluate treatment failure, Kaplan–Meier analysis and log-rank test were performed.

**Results:**

Overall, 98 PJI cases were assessed, including 33 with *Ab*-associated PJI and 65 with PJI involving other microorganisms (non–*Ab*-associated PJI). Independent RFs associated with *Ab*-associated PJI were revision arthroplasty [odds ratio (OR) = 3.01; 95% confidence interval (CI) = 1.15–7.90; *p* = 0.025] and nonelective arthroplasty (OR = 2.65; 95% CI = 1.01–7.01; *p* = 0.049). *Ab*-associated PJI was also more likely than non–*Ab*-associated PJI to be classified as a chronic late infection (OR = 5.81; 95% CI = 2.1–16.07; *p* = 0.001). *Ab*-associated PJI was not associated with treatment failure (*p* = 0.557).

**Conclusions:**

Late chronic infections, surgical revision and nonelective arthroplasty are well-known predictors of PJI but were also independently associated with *Ab*-associated PJI. Infections caused by *Ab* and surgical treatment with debridement, antibiotics and implant retention were not associated with PJI treatment failure.

**Trial registration:**

Study data supporting our results were registered with the Brazilian Registry of Clinical Trials (https://www.ensaiosclinicos.gov.br/rg/RBR-6ft5yb/), an open-access virtual platform for the registration of studies on humans performed in Brazil.

**Registration no.** RBR-6ft5yb.

**Supplementary Information:**

The online version contains supplementary material available at 10.1186/s12891-021-04393-4.

## Introduction

Worldwide, an increasing number of individuals have undergone joint-replacement surgeries, particularly at the hip and knee, either for elective reasons or following sustained trauma. Among all possible complications, prosthetic joint infection (PJI) is the one that is most feared; despite its low incidence, ranging from just 1 to 2% [1, 2] for primary and up to 4% for revision surgeries [3, 4], it boasts high morbidity and mortality rates. Gram-positive cocci (GPC), such as *Staphylococcus aureus* and coagulase-negative *Staphylococci*, are the major PJI-related microorganisms, followed by Gram-negative bacilli (GNB), with a prevalence ranging from 5 to 23% [4–6]. In some case series, PJIs attributed to GNB have been reported at rates greater than 40% [7, 8].

*Acinetobacter is a genus of Gram-negative bacteria including a total of 31 different species. Due to its ability to spread in health care environments, Acinetobacter baumannii (Ab) is currently the most difficult species to control and eradicate* [9]. This microorganism is ubiquitous in the environment [9] and has become one of the most successful pathogens associated with health care–related infections due to its ability to express a variety of antimicrobial resistance mechanisms and to form biofilms on both biotic and abiotic surfaces [10]. On a global scale, approximately 50% of *Ab* strains have been identified as multidrug-resistant (MDR). The World Health Organization (WHO) has declared carbapenem-resistant *Ab* in particular to be one of the most important species among *Enterococcus faecium, Staphylococcus aureus, Klebsiella pneumoniae, Ab, Pseudomonas aeruginosa* and *Enterobacter sp.*; these organisms are referred to collectively as the ESKAPE group and are considered priority pathogens due to the threat they pose to global public health, necessitating urgent actions and the development of new antibiotics to combat them [11, 12]. Unfortunately, in several Latin American countries, *Ab* strains have shown resistance to virtually all classes of antibiotics, including carbapenems*.* This worrisome microbial epidemiology has been identified at some Brazilian hospitals, where 77% of these isolates have exhibited resistance to carbapenems [13]. The production of oxacillin-hydrolysing carbapenemase (carbapenem-hydrolysing class D enzymes) has been identified as the most common antibiotic resistance mechanism, and the global dissemination of OXA-type clones, including OXA-23, OXA-72 and OXA-58, is regarded as the most common mechanism of antibiotic resistance [13, 14].

The emergence of musculoskeletal surgical site infections and orthopaedic implant–associated infections caused by *Ab* has become a matter of urgent concern for health care providers due to the limited therapeutic arsenal available, particularly against carbapenem-resistant strains [15]. Moreover, the treatment of PJIs caused by MDR and extensively drug-resistant (XDR) GNB, particularly *Ab*, is hampered by its ability to be encased within biofilms. The resistance of *Ab* against virtually all antimicrobials and its intrinsic capacity for biofilm formation may correlate with lower cure rates and increased disease morbidity since treatment usually requires a combination of highly toxic systemic antibiotics [16, 17]. Despite this challenge, to our knowledge, no published studies have investigated independent risk factors (RFs) for *Ab*-associated PJI. Indeed, few previous publications on *Ab*-associated PJI have attempted to describe, in a case series report format, aspects of surgical and antibiotic therapy [15, 18, 19]. Therefore, this study aimed to identify the independent RFs for *Ab-associated PJI* and to assess the role of *Ab* in treatment outcome.

## Materials and methods

### Study design

This study was performed as an observational, single-centre, retrospective, cohort study using data obtained from 2672 patients undergoing arthroplasties between January 2014 and July 2018, at a Brazilian orthopaedic referral centre. All patients diagnosed with PJI, either due to *Ab* (*Ab*-PJI) and other microorganisms (Non-*Ab*-PJI), were identified from clinical and microbiological records and surgical description sheets. The primary study endpoint was the identification of independent predisposing factors associated with PJI caused by *Ab* and secondary endpoint was to access if the *Ab-*PJI have influence on treatment outcome. The study included individuals aged 18 years or older who met the diagnosis criteria for PJI according to the Musculoskeletal Infection Society (MSIS) [20]. Inclusion criteria also required the same identified pathogen yielding in at least two peri-prosthetic tissue samples, and prospective follow-up period of a minimum one-year period. Patients who underwent arthroplasty at an institution other than ours, did not meet the criteria for PJI as defined by the MSIS or had culture-negative results were excluded. The study was reviewed and approved by the local ethics committee (approval no. 2,610,914 on April 20, 2018).

### Definitions

The PJI onset date was defined according to the date of the first observation of typical infectious signs and symptoms. MDR- *Ab* was defined as the nonsusceptibility of the identified pathogen to at least one antimicrobial agent from three or more different antimicrobial classes (e.g., aminoglycosides, cephalosporins with an anti-Pseudomonas effect, carbapenems, fluoroquinolones, penicillin + β-lactamase inhibitors, monobactams and polymyxin). *Ab* that were extensively drug-resistant (XDR) to multiple antibiotics were defined as those lacking susceptibility to at least one antimicrobial agent from all but two classes of antimicrobials [21].

Early-onset PJI was defined as those cases occurring < 3 months after the index surgery, whereas late PJI was defined as those cases in which the diagnosis occurred more than 3 months after the index surgery. The remission of infection was defined as the absence of clinical, laboratory, or radiological symptoms at the last medical follow-up (with a minimum follow-up time of 1 y). Therapeutic failure was defined as infection recurrence at a previously controlled site; requirement for new surgery, a second course of antimicrobial therapy, chronic antibiotic suppression, excision arthroplasty, or limb amputation; or death within the follow-up period [22, 23].

### Microbiological analysis

In the surgical ward, a minimal of three different periprosthetic tissue samples and synovial fluid were collected and processed for microbiology. Synovial fluid sample were aseptically inoculated into aerobic standard blood culture bottles. Tissue samples were homogenised in 3 ml of brain-heart infusion (BHI) broth for 1 min and inoculated onto aerobic sheep blood agar, chocolate agar, and anaerobic blood agar and into thioglycolate broth (BD Diagnostic Systems, Sparks, MD). The time limit for processing samples was 6 h. Aerobic were incubated aerobically at 35–37 °C in 5–7% CO_2_ for 7 days, and anaerobic plates were incubated at 37 °C for 14 days. Additionally, 0.5 ml of tissue homogenate was inoculated in thioglycolate broth, incubated for 14 days, and sub-cultured on blood agar plates when the broth became cloudy. Colonies of microorganisms growing on plates were identified, and their susceptibilities to antibiotics were tested according to standard microbiological techniques. The bacteria were identified by conventional biochemical and metabolic tests in accordance with the international standards and definitions established by the European Committee on Antimicrobial Susceptibility Testing (EUCAST) [24]**.** Sensitivity tests were performed using the disk diffusion technique, and the determination of minimum inhibitory concentrations (MICs) was performed by automated means or by the e-test method, the results of which are presented according to standardised microbiological techniques.

### Potential risk factors

Variables associated with the patient, surgery, and postoperative procedures were identified by reviewing the medical, intraoperative, and microbiological records to identify potential RFs for *Ab*-PJI. Demographic variables (sex and age), comorbidities (the presence and number of comorbidities, alcoholism, and smoking habits), the American Society of Anaesthesiologists (ASA) physical status classification, previous use of antibiotics during the past 3 m, and previous orthopaedic infections were assessed. Associated surgical aspects included the arthroplasty site (hip vs knee), total or partial arthroplasty, primary or revision surgery, and post-traumatic arthroplasty or elective arthroplasty. The factors related to the postoperative period that were considered included postoperative hematoma, the presence of sepsis at the time of diagnosis, concomitant infections diagnosed at different sites, and early or late infection. The surgical strategies, including debridement, antibiotics, and implant retention (DAIR) or any prosthesis removal (Non-DAIR), were assessed for survival and outcome analyses.

### Statistical analysis

For the overall study population and the groups defined as *Ab*-PJI and Non-*Ab*-PJI, qualitative variables are reported as the mean and percentage, and quantitative variables are presented as the median and standard deviation (SD). Associations between qualitative variables were analysed using the Chi-square test and Fisher’s exact test, as indicated. The associations between quantitative variables were assessed by logistic regression. The risk estimate was calculated for the associated variables and reported as the odds ratio (OR) with a 95% confidence interval (CI). The logistic regression model was used to select significant variables from among those identified as significant in univariate analyses. Only variables with significance less than 0.20 (*p* < 0.20) were included in the logistic regression. Variables with significance less than 0.05 (*p* < 0.05) in the multiple regression were included in the final model. To estimate the probability of survival as a function of time, Kaplan-Meier (KM) analyses were performed, and the resulting curves were compared using the log-rank method. All data were analysed using SPSS, version 23 (IBM-SPSS Inc., Chicago, IL, USA).

## Results

A total of 115 PJI cases were assessed for inclusion in the study; of these, 14 cases that did not meet the MSIS criteria for infection and three cases with less than two or with negative tissue cultures were excluded. Therefore, 98 PJI cases were finally analysed, including 33 in the *Ab*-associated PJI group and 65 in the non–*Ab*-associated PJI group.

### Study population

The demographic and clinical characteristics of the study population are summarised in Additional file 1. Most PJI patients were women (58.16%), with a mean ± standard deviation age of 67.3 ± 13.2 years. Interestingly, hip arthroplasty was the most frequent procedure (83.7%). More than 70% of patients had at least one comorbidity, with hypertension (61.2%) and diabetes mellitus (20.4%) being the most common. Arthroplasty was the primary surgery in 57.1% (56/98) of cases and was performed to address a fracture (nonelective) in 39.8% (39/98). PJI was classified as early in 69.4% (68/98), and 19.4% (19/98) of patients had experienced a PJI previously (Additional file 1).

### Microbiology

Among 33 patients with *Ab*-associated PJI, 27 strains were classified as XDR strains, four were classified as MDR strains and two strains were sensitive to multiple antibiotics. Although the rate of susceptibility to carbapenem was below 6%, all isolates were 100% susceptible to colistin (Table 1).

**Table 1:** Antibiotic susceptibility of *Ab* strains

Antibiotics	SPT	CIP	AMP/S	IMP	MER	PITA	CFP	AK	GM	CAZ	CO
Ab^a^. N (%)	8 24.24	1 3.03	5 15.15	1 3.03	2 6.06	1 3.03	0 0	15 45.45	5 15.15	0 0	33,100

*SPT* co-trimoxazole, *AMP/S* ampicillin-sulbactam, *CIP* ciprofloxacin, *IMP* imipenem, *MER* meropenem, *PITA* piperacillin/tazobactam, *CFP* cefepime, *AK* amikacin, *GM* gentamicin, *CAZ* ceftazidime, *CO* colistin

Culture yields in the non–*Ab*-associated PJI group were mainly *S. aureus*, *Enterobacter aerogenes* and *P. aeruginosa*. The rate of methicillin-resistant *S. aureus* identification was 7.0% (Additional file 2).

### Outcomes and potential risk factors for ab-associated PJI

During the univariate analysis, RFs associated with patient characteristics, surgery and the postoperative period were investigated for possible associations with *Ab*-associated PJI, as shown in Table 2. As compared with PJIs caused by other microorganisms, *Ab*-associated PJI was significantly associated with previous antibiotics usage in the last three months (51.5% vs 30.8%; *p* = 0.045), previous orthopaedic infections (36.4% vs 12.3%; *p* = 0.005), revision arthroplasty (60.6% vs 33.8%; *p* = 0.011) and posttraumatic/nonelective arthroplasty (54.4% vs 32.3%; *p* = 0.034). Eighteen patients in the A*b*-associated PJI group underwent nonelective arthroplasty for reasons such as high-energy trauma (*n* = 4) and falling from a height while elderly (*n* = 14). Patients with *Ab*-associated PJIs received more blood transfusions than those with PJIs caused by other microorganisms (36.4% vs 10.8%; *p* = 0.002). Early infections occurred in 48.5% (16/33) of patients with *Ab*-associated PJIs and 81.5% (53/65) of patients with non–*Ab*-associated PJIs (*p* = 0.001), respectively. Following an infectious diagnosis, DAIR was the surgical strategy chosen for 57.6% of the *Ab*-associated PJI cases (*p* = 0.047) (Table 2). The factors that remained independently associated with *Ab*-associated PJI following multiple logistic regression were revision arthroplasty [odds ratio (OR) = 3.01; 95% confidence interval (CI) = 1.15–7.90; *p* = 0.025] and nonelective arthroplasty (OR = 2.65; 95% CI = 1.01–7.01; *p* = 0.049). In addition, *Ab*-associated PJIs were more likely than non–*Ab*-associated PJIs to be classified as late chronic infections (OR = 5.81; 95% CI = 2.1–16.07; *p* = 0.001) (Table 3).

**Table 2:** Univariate analysis of risk factors associated with Ab-PJI^a^

Variables	Ab-PJI^a^ No. (%) ***N*** = 33	NON-Ab-PJI^**b**^ No. (%) ***N*** = 65	***P***-value^**#**^
**Demographic data** Age (median [variation]) (years)	71.0 (48–88)	69.0 (30–92)	0.626***
Females	16 (48.5)	41 (63.0)	0.166*
**Patient-related variables**			
Alcoholism	7 (21.2)	7 (10.77)	0.222*
Smoking	4 (12.1)	9 (13.8)	1.000*
Comorbidities (yes)	25 (75.8)	46 (70.8)	0.601**
SAH^c^	20 (60.6)	40 (61.5)	0.929**
DM^d^	8 (24.2)	12 (18.5)	0.502**
Malnutrition	4 (12.1)	4 (6.2)	0.436**
Anemia	2 (6.1)	0 (0.0)	0.111**
Cancer	0 (0.0)	1 (1.5)	1.000**
Lung disease	0 (0.0)	5 (7.7)	0.122**
Metabolic syndrome	5 (15.2)	13 (20.0)	0.558*
Cardiovascular disease	2 (6.1)	3 (4.6)	1.000**
Other comorbidities^e^	3 (9.1)	8 (12.3)	0.746**
**ASA score**^**f,** *^			
ASA 1	2 (6.1)	19 (29.2)	0.009**
ASA 2	22 (66.7)	25 (38.5)	
ASA 3	8 (24.2)	21 (32.3)	
ASA 4	1 (3.0)	0 (0.0)	
Use of antimicrobials^*^			
Previous use	17 (51.5)	20 (30.8)	0.045*
Single antimicrobial	6 (18.2)	5 (7.7)	0.104**
Antimicrobial combination	11 (33.3)	15 (23.1)	
Previous PJI	12 (36.4)	8 (12.3)	0.005*
Variables related to the surgical procedure^*^			
Arthroplasty			
Total	24 (72.7)	51 (78.5)	0.527*
Revision	20 (60.6)	22 (33.8)	0.011*
Non-elective	18 (54.4)	21 (32.3)	0.034*
Duration of the procedure > 2.5 h	3 (9.1)	12 (18.5)	0.223*
Blood transfusion	12 (36.4)	7 (10.8)	0.002*
Variables related to the postoperative period^*^			
Infection-related sepsis	2 (6.1)	0 (0)	0.111**
Concomitant non-orthopedic infection	18.2 (6)	10.8 (7)	0.352*
Surgical wound complications	42.4 (14)	26.6 (16)	0.071*
Early infection	48.5 (16)	81.5 (53)	0.001*
DAIR^g^	57.6 (19)	76.9 (50)	0.047*

*Ab-PJI*^a^ Prosthetic joint infection caused by *Acinetobacter baumannii*, *NON-Ab-PJI*^b^ non-*Acinetobacter* species causing prosthetic joint infection, *SAH*^c^ Systemic arterial hypertension, *DM*^*d*^ diabetes Mellitus; Other comorbidities^e^; rheumatoid arthritis, hypothyroidism, hyperthyroidism, depression; *ASA*^*f*^ American Anesthesiology Association, *DAIR*^*g*^ debridement, antibiotics and implant retention Significance probabilities refer to the Chi-squared test (*), Fisher’s exact test (**), and Student’s t-test (***)*P* values^#^ < 0.05 were considered statistically significant

**Table 3:** Predisposing factors independently associated to Acinetobacter baumannii PJI –multivariate analysis

Variables	***A. baumannii***^a^ No. (%) ***N*** = 33	Other bacteria No. (%) ***N*** = 65	Odds Ratio (OR) 95% CI	***P***-value^**a**^
Revision arthroplasty	20 (60.6)	22 (33.8)	3.01 (1.15–7.90)	0.025
Non-elective arthroplasty	18 (54.5)	21 (32.3)	2.65 (1.01–7.01)	0.049
Late infection	17 (51.5)	12 (18.5)	5.81 (2.1–16.07)	0.001

A. *baumannii*^a^: PJI caused by *Acinetobacter baumannii; CI* confidence interval

**Fig. 1:**
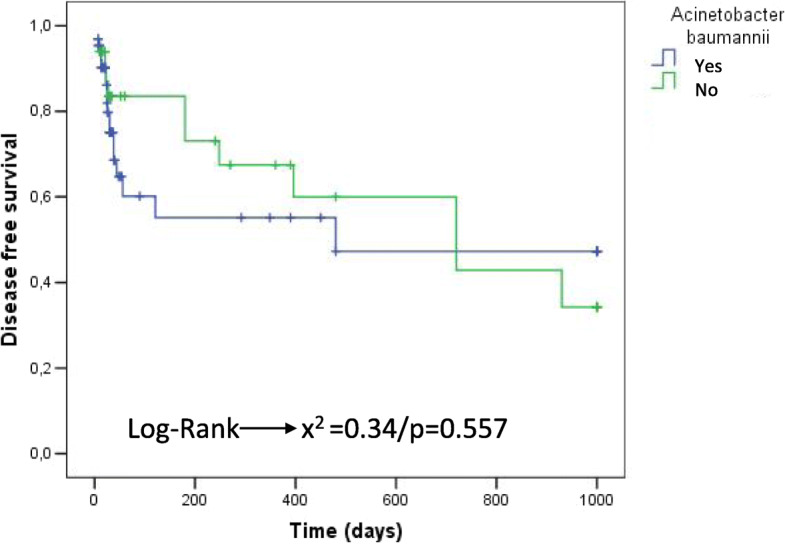
Kaplan–Meier survival curve for death/recurrence considering PJIs caused by *Ab*-PJI

**Fig. 2:**
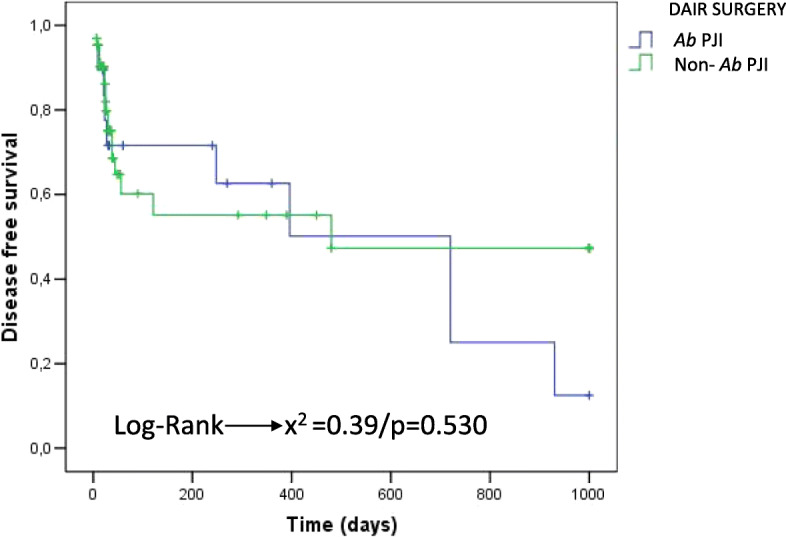
Kaplan-Meir survival curve for death/recurrence considerung PJI by *A. buamannii* PJI DAIR versus Non-*Ab*-PJI DAIR

On the Kaplan–Meier survival curve, infection by *Ab* was not identified as an RF for treatment failure (*p* = 0.557) (Fig. 1), and no increase in the failure rate was observed for the *Ab*-associated PJI group that underwent DAIR in comparison with the non–*Ab*-associated PJI group that underwent DAIR (*p* = 0.530) (Fig. 2).

## Discussion

To our knowledge, this is the first study to investigate predisposing factors associated with *Ab*-associated PJI. Interestingly the well-known predictors of PJI – which include revision surgeries, nonelective arthroplasties and late infections (PJI diagnosed after 3 m of index surgery) – were independently associated with *Ab* infection. This likely reflects the particular epidemiology of a Brazilian orthopaedic referral centre, where the rates of nosocomial SSI caused by MDR-GNB are high [25, 26]. In addition, the high selective pressure imposed by misuse of empirical broad-spectrum antibiotics is likely to have played a major role [27]. Since then, a local antimicrobial stewardship program has been implemented as a tool to enhance the appropriateness of antibiotics prescriptions.

In the microbiological sample, a greater frequency (81.8%) of *Ab* strains causing PJI were XDR strains, whereas 12.1% were MDR strains and only 6.1% were sensitive to multiple antibiotics. The rate of susceptibility to carbapenems was worryingly low, with only 3% of cases sensitive to imipenem and only 6% sensitive to meropenem. The higher prevalence of *Ab*-associated PJI at our institution was not assumed to represent an outbreak but instead an endemic nosocomial pathogen typically identified in the intensive care unit environment that boasts an overwhelming ability to colonise the human skin. Furthermore, before 2018, immediate postoperative care protocols for patients who undergo arthroplasty in our hospital were usually enacted in the intensive care unit, which is likely to have increased the rate of skin colonisation by *Ab* strains.

Orthopaedic implant–associated infections have traditionally been considered difficult to treat due to the formation of bacterial biofilms on the implant surface and the low levels of antibiotic penetration into bone tissue and biofilms [28, 29]. In addition, the higher levels of bacterial resistance commonly expressed by *Ab* makes treatment of infections involving this organism even more challenging due to the scarcity of available drugs and the potential for antibiotic-related toxicity [30]. Although *Ab* is ubiquitous in nature and colonises the skin of healthy individuals, most human infections by this organism are health care–associated. A systematic review by Falagas et al. [31], which included 55 articles describing *Ab* infections, linked *Ab* infections to prolonged hospital stays, intensive care unit treatments and the use of invasive devices.

Despite the poor availability of studies specifically describing *Ab*-associated PJI, *the number of osteomyelitis and fracture-related infections caused by Ab seems to be on the rise worldwide, especially when considering those associated with high-kinetic energy trauma and open fractures* *[*32]. *Many studies have described a strong association between complex traumatic gunshot wounds resulting in fracture-related infections or osteomyelitis and Ab infection in various conflict-affected regions, such as Iraq, Afghanistan and Yemen* *[*32–35]. However, whether *Ab* is acquired during the act of injury itself from primary contamination or is acquired in the hospital during the trauma care and subsequent surgical procedures remains unclear. In addition to reports from Middle Eastern countries, a study by Vanegas et al. [36] from Colombia addressed osteomyelitis, skin and soft tissue infections and reported an increased number of infections caused by *Ab,* with a strong association with recent hospitalisation or surgery and previous use of antimicrobials in the past 6 m. Despite the increased risk of PJI following revision surgery [37–39], the association between revision arthroplasty and *Ab*-associated PJI has not yet been explored in the literature. However, we are aware that implant contamination during surgery is a primary source of infection, and patients previously colonised with *Ab* may be at increased risk for PJI.

In our study, a 2.6-fold increase in the risk of *Ab*-associated PJI was identified among patients undergoing an emergency arthroplasty, which suggests that, similar to in the case of fracture-related infections, posttraumatic arthroplasty may be a factor that predisposes patients to *Ab*-associated PJI. The reasons underlying the association between trauma and *Ab* infection were not elucidated in this study and require further investigation. In addition, we were unable to identify any independent associations between *Ab*-associated PJI and closed proximal femoral fractures in the elderly population, recent hospitalisation history or recent use of antibiotics. However, nonelective arthroplasties may necessitate longer preoperative hospital stays due to the mandatory propaedeutic for assessing preoperative risks and the need to compensate for clinical comorbidities prior to performing the surgical procedure, which might increase the risk of colonisation by *Ab* [40]. However, the length of preoperative hospital stay, which could validate this hypothesis, was not a variable that was assessed in the present study.

In our study, late PJI was independently associated with *Ab* infection, and a possible explanation may rely upon the lower level of virulence expression when bacteria express multiple antibiotic-resistant mechanisms. Several mechanisms associated with antimicrobial resistance, including pump efflux and biofilm organisation abilities, also reduce the bacterial replicative capacity [41]. This stationary phase, associated with biofilm formation and maturation, is likely to cause *Ab* infections to develop more slowly, increasing the likelihood of being diagnosed as late PJI [42, 43]. Neither *Ab* infection nor the surgical strategy used after the infectious diagnosis was independently associated with the final outcome or the risk of treatment failure. Few studies to date have assessed the prognostic factors associated with *Ab*-associated PJI development, although some have reported high rates of therapeutic failure in *Ab*-associated PJIs; for example, Vasso et al. [19] described a 33.3% failure rate for the treatment of *Ab*-associated PJI. However, their study was underpowered, assessing only nine patients in a group containing a mix of infections associated with both *Ab* and *P. aeruginosa*. Another study that assessed the outcomes of PJI caused by GNB -MDR reported that infections caused by MDR/XDR GNB were associated with high therapeutic failure rates when DAIR (52.2%) was performed as compared to when non-DAIR treatment strategies were employed (23.4%) [44]. However, only three patients had *Ab*-associated PJIs in this previous study, which is not a representative sample.

Importantly, the present study has potential limitations. First, it was performed as a retrospective investigation and took place at a single centre located in a major city in a developing country offering specialised orthopaedic care for the regional population. Consequently, the results obtained at our hospital may not apply to other hospitals. In addition, the identification and sensitivity tests were performed using nonautomated methods, and no molecular or genotypic analyses were performed to identify clonal variants or similar patterns of resistance mechanisms. Furthermore, no pairing was performed between the *Ab*-associated PJI and non–*Ab*-associated PJI groups to control for preoperative hospitalisation times or preoperative colonisation by *Ab,* which could support the hypothesis that PJI contamination occurred intraoperatively. However, this study explored the previously unexamined issue of PJI-predisposing factors and relied on the largest number of *Ab* infection cases described to date, with a high frequency of MDR/ XDR strains.

## Conclusions

The findings suggest that nonelective and revision arthroplasties primarily performed due to trauma and PJI diagnosed three months after the index surgery were independently associated with *Ab*-associated PJI. In addition, PJI caused by *Ab* was not associated with treatment failure, and no difference according to DAIR in the A*b*-associated PJI versus non–A*b* PJI groups was identified for the disease-free survival rate. The present study adds relevant data to the growing field of MDR-GNB PJI cases, but larger, multicentre cohort studies are still needed.

## Supplementary Information

**Additional file 1:.** Demographics and clinical characteristics of study population

**Additional file 2:.** Microbiological description of the NON-Ab-PJI group

## Data Availability

Study data supporting our results were registered at an open access virtual platform for registration of studies on humans performed in Brazil. The Brazilian Registry of Clinical Trials (ReBEC) http://www.ensaiosclinicos.gov.br/rg/RBR-6ft5yb/ Register Number: RBR-6ft5yb.

## Abbreviations

*Ab*: *Acinetobacter baumannii*
ASA: American Society of Anesthesiologists
BHI: Brain-heart infusion
CI: Confidence interval
DAIR: (Debridement, antibiotics, and implant retention)
EUCAST: European Committee on Antimicrobial Susceptibility Testing
GNB: Gram-negative bacilli
GPC: Gram-positive cocci
ICU: Intensive care unit
KM: Kaplan–Meier
OR: Odds ratio
OXA: Oxacillin-hydrolysing
MRSA: Methicillin-resistant *Staphylococcus aureus*
MIC: Minimum inhibitory concentrations
MDR: Multidrug-resistant
MSIS: Musculoskeletal Infection Society
PJI: Prosthetic joint infection
RF: Risk factors
SD: Standard deviation
SSI: Surgical site infections
WHO: World Health Organization
XDR: Extensively drug-resistant
