# Supplementary material for: Non-elective and revision arthroplasty are independently associated with hip and knee prosthetic joint infection caused by Acinetobacter baumannii: a Brazilian single center observational cohort study of 98 patients
Source: BMC Musculoskelet Disord. 2021 Jun 2;22:511. doi: 10.1186/s12891-021-04393-4 (PMC8173725; doi:10.1186/s12891-021-04393-4)
Supplement: Supplementary file 1 — Additional file 1:. Demographics and clinical characteristics of study population [file 12891_2021_4393_MOESM1_ESM.docx]

**Additional file 1:** Demographics and clinical characteristics of study population.
