# Supplementary material for: Non-elective and revision arthroplasty are independently associated with hip and knee prosthetic joint infection caused by Acinetobacter baumannii: a Brazilian single center observational cohort study of 98 patients
Source: BMC Musculoskelet Disord. 2021 Jun 2;22:511. doi: 10.1186/s12891-021-04393-4 (PMC8173725; doi:10.1186/s12891-021-04393-4)
Supplement: Supplementary file 2 — Additional file 2:. Microbiological description of the NON-Ab-PJI group [file 12891_2021_4393_MOESM2_ESM.docx]

**Additional file 2:** Microbiological description of the NON-Ab-PJI group.

| **Microorganisms**  **Number of Samples** | **NON-Ab-PJI^a^**  **No. (%)**  **N= 71** |
| --- | --- |
| MSSA^b^ | 16 (22.5) |
| *Enterobacter aerogenes* | 12 (16.9) |
| *Pseudomonas aeruginosa* | 9 (12.7) |
| *Klebsiella pneumoniae* | 8 (11.3) |
| *Proteus mirabilis* | 7 (10.8) |
| *Escherichia coli* | 6 (8.5) |
| MRSA^c^ | 5 (7.0) |
| *Proteus vulgaris* | 3 (4.2) |
| *Morganella morgannii* | 2 (2.8) |
| *Enterobacter cloacae* | 2 (2.8) |
| *Enterobacter sakazakii* | 1 (1.4) |

NON-Ab-PJI^a^: non-*Acinetobacter* species causing prosthetic joint infection; MSSA^b^: Methicillin-sensitive *Staphylococcus aureus;* MRSA^c^ Methicillin-resistant *Staphylococcus aureus.*
